# Cerebral Effects of Commonly Used Vasopressor-Inotropes: A Study in Newborn Piglets

**DOI:** 10.1371/journal.pone.0063069

**Published:** 2013-05-20

**Authors:** Gitte H. Hahn, Simon Hyttel-Sorensen, Sandra M. Petersen, Ole Pryds, Gorm Greisen

**Affiliations:** 1 Department of Neonatology, Copenhagen University Hospital–Rigshospitalet, Copenhagen, Denmark; 2 Department of Pediatrics, Copenhagen University Hospital–Hvidovre Hospital, Hvidovre, Denmark; Idaho State University, United States of America

## Abstract

**Background:**

Despite widespread use in sick infants, it is still debated whether vasopressor-inotropes have direct cerebral effects that might affect neurological outcome. We aimed to test direct cerebrovascular effects of three commonly used vasopressor-inotropes (adrenaline, dopamine and noradrenaline) by comparing the responses to those of nonpharmacologically induced increases in blood pressure. We also searched for reasons for a mismatch between the response in perfusion and oxygenation.

**Methods:**

Twenty-four piglets had long and short infusions of the three vasopressor-inotropes titrated to raise mean arterial blood pressure (MAP) 10 mmHg in random order. Nonpharmacological increases in MAP were induced by inflation of a balloon in the descending aorta. We measured cerebral oxygenation (near-infrared spectroscopy), perfusion (laser-Doppler), oxygen consumption (co-oximetry of arterial and superior sagittal sinus blood), and microvascular heterogeneity (side stream dark field video microscopy).

**Results:**

Vasopressor-inotropes increased cerebral oxygenation significantly less (p≤0.01) compared to non-pharmacological MAP increases, whereas perfusion was similar. Furthermore, cerebral total hemoglobin concentration increased significantly less during vasopressor-inotrope infusions (p = 0.001). These physiologic responses were identical between the three vasopressor-inotropes (p>0.05). Furthermore, they induced a mild, although insignificant increase in cerebral metabolism and microvascular heterogeneity (p>0.05). Removal of the scalp tissue did not influence the mismatch (p>0.05).

**Conclusion:**

We demonstrated a moderate vasopressor-inotrope induced mismatch between cerebral perfusion and oxygenation. Scalp removal did not affect this mismatch, why vasopressor-inotropes appear to have direct cerebral actions. The statistically nonsignificant increases in cerebral metabolism and/or microvascular heterogeneity may explain the mismatch. Alternatively, it may simply reflect a vasopressor-inotrope-induced decrease in the arterial-to-venous volume ratio as detected by near-infrared spectroscopy.

## Introduction

Despite widespread use of antihypotensive therapy in sick newborn infants, controversy still exists as to whether adverse neurological outcome is caused by the antihypotensive therapy itself, by inappropriate administration of the therapy causing sudden alterations in the blood pressure or by the severity of the disease [1]–[4]. Since premature [5] and sick infants [6] have increased blood-brain barrier permeability, direct cerebrovascular as well as cerebral effects of antihypotensive therapy seem possible [7]. Contribution of adrenergic mechanisms to cerebrovascular regulation may be relatively unique to the developing brain [8], [9]. Thus, understanding how commonly used vasopressor-inotropes affect cerebrovascular mechanisms is of major clinical relevance in newborn infants.

Recently, an experimental study by our group suggested that dopamine, the most frequently used vasopressor-inotrope in sick infants, induces an unexplained mismatch between cerebral perfusion and oxygenation [10]. Based on this finding we set out to investigate how three commonly used vasopressor-inotropes - adrenalin, dopamine and noradrenalin – might affect cerebral perfusion and oxygenation. Besides, we evaluated whether any mismatch could be attributable to (i) increased cerebral metabolism [11], [12], (ii) increased microvascular heterogeneity [13] or simply (iii) vasoconstriction in extracranial blood vessels contaminating the cerebral oxygenation-signal, thereby making near-infrared spectroscopy (NIRS) inappropriate to monitor preservation of cerebral oxygenation as a surrogate measure of cerebral blood flow during antihypotensive therapy in newborn infants [14].

## Materials and Methods

The Danish Animal Experiments Inspectorate approved the experimental protocol (approval ID: 2009/561-1723).

### Anesthesia and Monitoring

In 24 newborn piglets (median age: 1 day, range: 1–6 days; median weight: 1.7 kg, range: 1.3–2.1 kg) preanesthesia was induced by 3% isoflurane supplemented with an intravenous induction dose of propofol (5 mg/kg) and fentanyl (20 µg/kg). Subsequently, isoflurane inhalation was discontinued while intravenous propofol (15 mg/kg/h) was maintained throughout the experiment. Mechanical ventilation was instituted after tracheostomy and ventilator settings were adjusted to keep arterial saturation above 95% and arterial tension of CO_2_ between 4 to 6 kPa. Arterial oxygen saturation and pulse rate was monitored by pulse oximetry from the forepaw (Radical 7, Masimo, Hannover, Germany). Piglets were kept on a warming pad to maintain rectal temperature at 38.5–39.5°C.

### Surgical Preparation

The femoral arteries were cannulated with: (*i*) a pressure and blood gas monitoring line and (*ii*) a 4 Fr catheter with an inflatable balloon near its tip (Fogarty Fortis, Arterial Embolectomy Catheter, Edwards Lifesciences, USA). Inflation of this balloon occluded the lumen of the thoracic part of aorta resulting in a nonpharmacologically induced increase in blood pressure in the upper body. The femoral vein was cannulated with a central venous line (4 Fr double lumen catheter) for drug and fluid infusion (glucose 10%, 2.5 ml/kg/h). A craniotomy was performed in the right parietal region with a biopsy punch (diameter: 5 mm) for dural placement of a laser-doppler flowmetry (LDF) probe. In half of the piglets (n = 12) yet another transdural craniotomy was performed in the left parietal region for video microscopic measurements (diameter: 15 mm). This transdural craniotomy was protected by wet gauzes and desiccation was prevented by local administration of 2 ml saline solution (37°C) each quarter of an hour. Finally, a cannula (22 G) was inserted at the bregma into the sagittal sinus to permit repeated sampling of cerebral venous blood. Piglets recovered for 0.5 h after the surgical procedures.

All piglets received antibiotics (ampicillin: 200 mg/kg; gentamicin: 4 mg/kg) and saline (10 ml/kg) as a single dose prior to surgical preparation. All incisions were infiltrated with bupivacaine (2.5 mg/ml).

### Experimental Protocol

**Figure 1:**
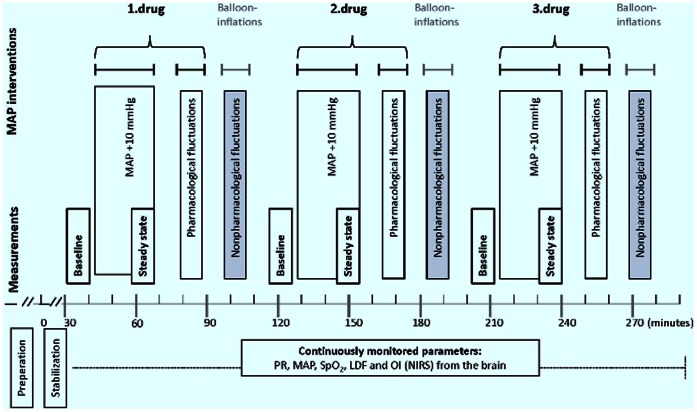
The experimental protocol. Baseline and steady state measurements included duplicate blood samples for metabolic measurements in all piglets and video microscopic examination of the cortical microvascular heterogeneity in of the 12 piglets. Blood pressure fluctuations were induced pharmacologically and nonpharmacologically to evaluate a possible direct drug induced effect on cerebral perfusion and oxygenation. PR, pulse rate; MAP, mean arterial blood pressure; SaO_2_, arterial oxygen saturation; LDF, laser Doppler flow; OI, oxygenation index; NIRS, near infrared spectroscopy.

**Figure 2:**
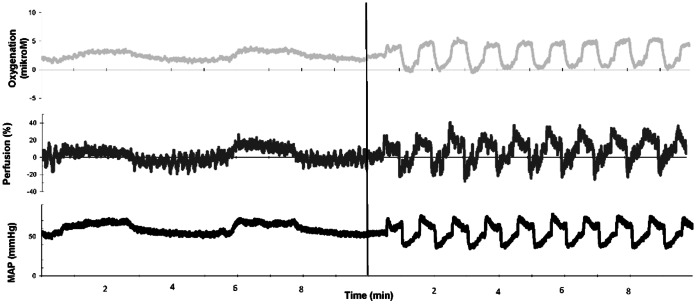
Short infusions of the vasopressors. Example of pharmacologically (first 10 min) and nonpharmacologically (last 10 min) induced changes in mean arterial blood pressure (MAP) and corresponding changes in cerebral perfusion (LDF in % from baseline) and oxygenation (OI measured with near infrared spectroscopy). Pharmacologically induced changes consisted of two repeated infusions lasting 2½ minute, interspaced with pauses lasting 2½ minute to reach baseline levels. This was followed by nonpharmacological MAP fluctuations induced by balloon inflations: the thoracic aorta balloon was inflated 10 times, and inflations lasted 30 sec interspaced by 30 sec pauses. The vertical line represents a 10 min pause between the induced changes in MAP.

We used long and short infusions of the vasopressor-inotropes (adrenalin, dopamine and noradrenalin) titrated to raise mean arterial blood pressure (MAP) 10 mmHg as well as nonpharmacologically induced changes in MAP (Fig. 1 and 2). The long infusions were applied to study steady state responses and effect on cerebral metabolism and microvascular heterogeneity. The short repeated infusions were applied to differentiate the cerebral effects of vasopressor-inotropes from cerebral autoregulatory effects elicited by the systemic effect of the drugs. Nonpharmacologically induced changes in MAP were induced by repeated inflations of the thoracic balloon. The balloon was inflated 10 times, and inflations lasted 30 sec interspaced by 30 sec pauses (Fig. 2). Piglets rested for 10 min between each MAP intervention to ensure adequate washout/return to baseline. Thirty minutes after the drug infusion was stopped, the next drug was tested in the same way. Between each drug, a volume of six times the estimated catheter dead space (0.2 ml) was withdrawn from the catheter to prevent mixture of drug effects.

The vasopressor-inotropes were tested in randomized and balanced order. The investigators were blinded to this randomization until end of data analysis.

Pulse rate, arterial oxygen saturation, MAP, cortical laser-doppler flow (LDF) and cerebral oxygenation were sampled continuously throughout the experiment (sampling rate: 2 Hz).

### Cerebral Perfusion and Oxygenation

LDF monitored cerebral perfusion (Perimed 5010, Stockholm, Sweden). The probe was placed in a metal washer glued to the skull. LDF measures changes in red blood cell velocity in non-absolute values. Consequently, LDF values are expressed as percentage change compared to values averaged over the last minute before each intervention.

Near-infrared spectroscopy (NIRS) monitored cerebral oxygenation (NIRO-300 spectrophotometer, Hamamatsu Photonics, Hamamatsu City, Japan). The probes were fixed in a nontransparent, soft probe holder (interoptode distance: 4 cm) and placed on the skin. In 12 of the piglets (those who did not undergo video microscopic examination of microvascular heterogeneity), a second NIRS probe was placed directly on the skull to estimate a possible extracranial contribution to the NIRS-signal. Changes in the concentrations of oxygenated and deoxygenated hemoglobin were recorded and used to calculate the oxygenation index (OI). OI is the difference between oxygenated and deoxygenated hemoglobin divided by a factor of 2. It reflects changes in cerebral blood flow (CBF), provided constant levels of SaO_2_ and cerebral metabolism [15]. The summation of oxygenated and deoxygenated hemoglobin represents the change in total hemoglobin (tHb), which reflects the change in cerebral blood volume within the illuminated area.

During the dynamic responses, i.e. during the short repeated infusions and the nonpharmacologically induced changes in MAP, frequency analysis yielding gain between MAP and percentage change in LDF (gain-LDF), OI (gain-OI) and tHb (gain-tHb) were computed for frequencies between 0.003 and 0.04 Hz (fluctuation time: 25–300 sec) (Matlab, Math Works, Natick, Maasachusetts). Gain measures change in output signal (here LDF, tHb and OI) per change in input signal (here MAP). Each 10-minute epoch of data was subdivided into three segments of 5 minutes with 50% overlap. A Hanning-window was applied to minimize spectral leakage. Both time-signals were then detrended and transformed into power spectrum densities by Fourier transformation (Matlab, MathWorks, Natick, MA).

Discerning the possible direct actions of vasopressor-inotropes on the cerebral circulation is complicated by their additional effects on the systemic circulation. Therefore, we compared gain during short repeated vasopressor-inotrope infusions and gain during nonpharmacologically induced changes in MAP to account for a possible indirect effect of cerebral autoregulation on cerebral vasculature elicited by a vasopressor-inotrope induced increase in MAP.

### Cerebral Metabolism

Venous (superior sagittal sinus) and arterial blood samples were obtained simultaneously and analyzed immediately (Radiometer, Copenhagen, Denmark). We estimated the cerebral arteriovenous oxygen content difference (AVDO_2_) according to the following formula:
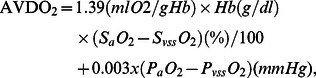
where Hb is hemoglobin, 1.39 is the oxygen carrying capacity of Hb, S_a_O_2_ is the arterial oxygen saturation, S_vss_O_2_ is the venous oxygen saturation in sinus sagittalis, P_a_O_2_ is the arterial oxygen tension, P_vss_O_2_ is the venous oxygen tension in sinus sagittalis, and 0.003 is the solubility of oxygen in blood. We used the mean of duplicate measurements to characterize AVDO_2_ in each state. Cerebral metabolic rate of oxygen (CMRO_2_) was calculated in relative values based on percentage change in LDF between baseline and steady state:



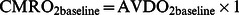


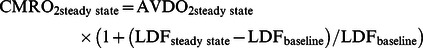



### Cerebral Microcirculation

We used a side stream dark field video microscope (Microscan, MicroVision Medical Inc, Amsterdam, Netherlands) to evaluate possible alterations in cerebral microcirculatory heterogeneity. Basically, this is a handheld device that illuminates an area of 1.08×0.81 mm and penetrates 0.9 mm with light in a wavelength (580 nm) that is absorbed by red blood cells (RBC). In this way RBCs appear dark and visualize microcirculatory flow. Image acquisition and subsequent analyses were performed according to published consensus criteria [16]. In brief, the lens of the imaging device was applied to the cerebral cortex, and absence of pressure artifacts was secured by preservation of flow in large vessels. Within the craniotomy, three recordings of 25 sec. each were recorded from different regions during both baseline and steady state. To minimize possible bias introduced by subjective flow classification, one investigator (S.M.P), who had no involvement in the data collection, performed all analyses. Furthermore, recordings were coded randomly and analyzed by means of a validated software package (Automated Vascular Analysis; AVA 3.0, MicroVision medical, Amsterdam, Netherlands) [17]. The software performs automatic vessel recognition and measurement (length and diameter), whereas flow is classified by eye. We used proportion of perfused vessels (PPV; proportion of vessels with continuous or sluggish flow) to determine microvascular heterogeneity with regard to (*i*) anatomical distribution and (*ii*) vessel type. Anatomical heterogeneity index was calculated as the difference between the highest and lowest value of PPV divided by the mean of the three different regions investigated [18]. This index was used to investigate whether vasopressor-inotropes increased flow in some areas but not in others. Vessel type heterogeneity index was calculated as PPV in capillaries (diameter <25µm) divided by PPV in all vessels. This index was used to investigate whether vasopressor-inotropess affected capillary flow differentially.

### Statistics

Parametric (paired Student’s t-test) or non-parametric (Wilcoxon Signed Rank Test) statistical methods were used as appropriate to test paired differences between baseline and steady state as well as between pharmacologically and nonpharmacologically induced MAP-changes. One-way repeated measures ANOVA was used to compare the cerebrovascular effects between the drugs and to investigate if gain during nonpharmacologically induced changes in MAP - as a measure of cerebral autoregulation – changed over time. A two-tailed p-value <0.05 was considered significant (SPSS statistics 17.0). Continuous data are reported in median [25^th^–75^th^]. Because of insufficient signal quality, one piglet was excluded from the study (n = 23), leaving 11 piglets with video microscopic examinations.

## Results

A median adrenaline dose of 0.6 [0.5–0.7] µg per kg pr min was used to increase MAP with 8 [6]–[11] mmHg. The equivalent dosages and responses were 25 [16]–[31] µg per kg pr min and 11 [6]–[13] mmHg for dopamine and 0.4 [0.4–0.4] µg per kg pr min and 8 [5]–[10] mmHg for noradrenaline. All three vasopressor-inotropes increased MAP significantly from baseline (p<0.001) (Table 1). From a cerebral hemodynamic point of view, the piglets stayed stable during the experiment, as their cerebral autoregulatory capacity – measured by means of gain-LDF and gain-OI during nonpharmacologically induced changes in MAP – did not change significantly during the experiment (p = 0.2 and p = 0.5, respectively).

**Table 1:** Steady state responses during vasopressor infusion.

Vasopressor		Baseline		Steady state		p-value
**Adrenaline**						
mean arterial blood pressure	(mmHg)	63	[54–69]	71	[62–73]	<0.001*
cerebral perfusion	(percent of baseline)	1		1.18	[1.02–1.5]	0.002*
cerebral oxygenation	(µM )	0.4	[−0.9–3.0]	2.1	[−0.7–5.3]	0.06
CMRO_2_	(relative values)	5.3	[4.4–6.7]	5.5	[4.0–6.9]	0.9
anatomical heterogeneity index						
capillaries		0.22	[0.16–0.39]	0.27	[0.11–0.47]	0.4
all		0.14	[0.12–0.23]	0.06	[0.04–0.13]	0.006*
vessel type heterogeneity index		0.77	[0.72–0.87]	0.74	[0.72–0.80]	0.2
**Dopamine**						
mean arterial blood pressure	(mmHg)	58	[49–65]	66	[57–77]	<0.001*
cerebral perfusion	(percent of baseline)	1		1.15	[1.00–1.2]	0.001*
cerebral oxygenation	(µM )	1.0	[−0.4–3.4]	2.7	[−0.3–6.5]	0.004*
CMRO_2_	(relative values)	6.1	[5.1–7.2]	6.1	[4.7–7.0]	0.7
anatomical heterogeneity index						
capillaries		0.12	[0.10–0.32]	0.25	[0.10–0.44]	0.7
all		0.09	[0.08–0.16]	0.11	[0.05–0.23]	0.5
vessel type heterogeneity index		0.80	[0.75–0.81]	0.71	[0.64–0.80]	0.3
**Noradrenaline**						
mean arterial blood pressure	(mmHg)	58	[49–68]	67	[56–74]	<0.001*
cerebral perfusion	(percent of baseline)	1		1.02	[0.98–1.10]	0.07
cerebral oxygenation	(µM )	0.4	[−1.1–3.4]	1.7	[0.4–3.5]	0.01*
CMRO_2_	(relative values)	5.5	[3.8–7.9]	5.9	[4.5–7.8]	0.1
anatomical heterogeneity index						
capillaries		0.20	[0.12–0.32]	0.22	[0.12–0.28]	0.9
all		0.09	[0.06–0.17]	0.14	[0.06–0.22]	0.3
vessel type heterogeneity index		0.80	[0.71–0.82]	0.78	[0.68–0.85]	0.6

Values are reported in median [25^th^–75^th^].

During steady state, where additional effects on the systemic circulation were not accounted for, dopamine increased both cerebral perfusion and oxygenation significantly (p = 0.001 and p = 0.004, respectively). Adrenaline increased cerebral perfusion significantly (p = 0.002), whereas the increase in cerebral oxygenation did not reach significance (p = 0.06). Noradrenaline did not increase cerebral perfusion significantly (p = 0.07), whereas cerebral oxygenation increased significantly (p = 0.01) (Table 1). These responses, however, did not differ significantly among the vasopressor-inotropes (ANOVA p = 0.053 for perfusion and p = 0.7 for oxygenation) (Table 2).

**Table 2:** Comparison of the effects of adrenaline, dopamine and noradrenaline on cerebral perfusion and oxygenation.

	N	Adrenaline		Dopamine		Noradrenaline		p-value
**Steady state**								
cerebral perfusion (%LDF change pr. mmHg)	23	2.2	[0.1–6.0]	1.4	[−0.2–3.8]	0.4	[−0.1–1.2]	0.053
cerebral oxygenation (µM pr. mmHg)	23	0.10	[−0.04–0.38]	0.13	[0.07–0.37]	0.10	[0.01–0.18]	0.7
**Repeated infusions**								
cerebral perfusion (gain PU; % pr. mmHg)	23	1.2	[0.8–1.4]	1.4	[1.0–1.9]	1.3	[0.9–1.7]	0.5
cerebral oxygenation (gain OI; µM pr. mmHg)	23	0.08	[0.04–0.12]	0.07	[0.05–0,13]	0.06	[0.05–0.11]	0.8

Values are reported in median [25^th^–75^th^].

To level out a possible masking effect of cerebral autoregulation on the cerebral response to vasopressor-inotrope infusion, we compared gain-LDF and gain-OI between pharmacologically and nonpharmacologically induced changes in MAP. Generally, we observed a mismatch between cerebral perfusion (gain-LDF) and oxygenation (gain-OI), as cerebral perfusion was similar while cerebral oxygenation was significantly lower, except for adrenaline, where the increase in cerebral perfusion was significantly lower compared to nonpharmacologically induced changes in MAP (Table 3). Again, however, these responses did not differ significantly among the vasopressor-inotropes (ANOVA: p = 0.5 and p = 0.8, for cerebral perfusion and oxygenation respectively) (Table 2).

**Table 3:** Cerebral responses during short repeated infusions.

Vasopressor		nonpharmacological intervention		pharmacological intervention		p-value
		(N = 23)		(N = 23)		
**Adrenaline**						
Cerebral perfusion	(gain-LDF; % pr.mmHg)	1.3	[1.1–1.6]	1.2	[0.8–1.4]	0.02*
cerebral oxygenation	(gain-OI; µM pr.mmHg)	0.11	[0.09–0.15]	0.08	[0.04–0.12]	0.01*
**Dopamine**						
Cerebral perfusion	(gain-LDF; % pr.mmHg)	1.4	[1.1–1.7]	1.3	[0.9–1.7]	0.4
cerebral oxygenation	(gain-OI; µM pr.mmHg)	0.12	[0.08–0.15]	0.07	[0.05–0.13]	0.01*
**Noradrenaline**						
Cerebral perfusion	(gain-LDF; % pr.mmHg)	1.5	[1.1–1.9]	1.3	[0.9–1.7]	0.1
cerebral oxygenation	(gain-OI; µM pr.mmHg)	0.11	[0.08–0.14]	0.06	[0.05–0.11]	0.001*

Response in cerebral perfusion and oxygenation during repeated vasopressor infusions (pharmacological intervention) compared to nonpharmacological intervention (balloon inflations). Values are reported in median [25^th^–75^th^].

Gain-tHb was significantly lower during vasopressor-inotrope infusions compared to non-pharmacologically induced changes in MAP, 0.09 [0.05–0.10]µM/mmHg and 0.11 [0.07–0.13] µM/mmHg, respectively (p = 0.001). The response did not differ significantly among the three vasopressor-inotropes (p>0.05). These numbers indicate an increase in cerebral blood volume during both pharmacologically and nonpharmacologically induced changes in MAP.

CMRO_2_ was not affected significantly by any of the vasopressor-inotropes, although we observed a mild increase in CMRO_2_. Likewise, we demonstrated an insignificant increase in microvascular heterogeneity during vasopressor-inotrope infusion. The anatomical heterogeneity index increased, and the vessel type heterogeneity index decreased, indicating a minor hypoperfusion (i) in some areas and (ii) in capillaries compared to all vessels, respectively (Table 1).

Cutaneous vasoconstriction did not influence the NIRS measurement of cerebral oxygenation. During steady state, the change in cerebral oxygenation was 1.03 [−0.01–2.09] µM, when the NIRS probe was placed on the skin, compared to 0.95 [−0.25–1.85], when the probe was placed directly on the skull (p = 0.053). Also, during short repeated infusions, we did not observe any differences: (0.07 [0.05–0.11] µM per mmHg on the skin and 0.06 [0.04–0.07] µM per mmHg on the skull; p = 0.2).

## Discussion

We tested how three commonly used vasopressor-inotropes – adrenaline, dopamine and noradrenaline - affect cerebral perfusion, oxygenation, metabolism, and microvascular heterogeneity in newborn piglets. To the best of our knowledge, we are first to investigate possible changes in cerebral microvascular heterogeneity during antihypotensive therapy. Also, we are first to test whether cerebral oxygenation, as measured with NIRS, is influenced by cutaneous vasoconstriction, and whether CMRO_2_ is affected during vasopressor-inotrope therapy in the newborn brain.

The direct effect of vasopressor-inotropes on the cerebral circulation remains contentious, and clinical studies in infants are conflicting. Besides different methods of measurements of cerebrovascular responses, the main cause of disagreement seems to be variability of the study population with a varying degree of (*i*) hypotension, (ii) impaired cerebral autoregulation and (*iii*) integrity of the blood-brain barrier. Cerebral autoregulation, the myogenic response that ensures a fairly constant cerebral blood flow within a wide range of arterial blood pressures, may be impaired in hypotensive infants [19]–[21]. This explains, why dopamine increases cerebral blood flow in hypotensive [19], [22]–[24] but not in normotensive infants [25], [26]. Thus, to study a direct cerebral effect of vasopressor-inotropes, the myogenic component of cerebral autoregulation needs to be accounted for. In our study this was accomplished by contrasting the cerebral effect of pharmacologically and nonpharmacologically induced changes in blood pressure. In line with an earlier study by our group [10], we found a mismatch between cerebral perfusion and oxygenation, as oxygenation increased less than perfusion after administration of vasopressor-inotropes. Removing the scalp did not matter, so vasopressor-inotropes appear to have direct cerebral actions.

From an overall point of view, the three vasopressor-inotropes did not affect cerebral oxygenation or perfusion differently. Looking at individual differences during steady state, however, adrenaline apparently increased cerebral perfusion to a larger extent than dopamine and noradrenaline. This observation was not consistent with steady state changes in oxygenation or changes in perfusion or oxygenation during repeated infusions, where individual responses did not differ (Table 2). Therefore, we do not attach significance to this single observation.

The fact that cerebral oxygenation did not depend on whether the NIRS probe was placed on the skin or directly on the skull indicates that NIRS can be used to monitor preservation of cerebral oxygenation as a surrogate measure of cerebral blood flow during vasopressor-inotrope therapy. Contrary to our finding, experiments in adults generally report a significant extracranial contamination of the NIRS signal [14], which complicates the use of NIRS to monitor preservation of cerebral oxygenation during vasopressor-inotrope treatment [27]. Differences in thickness of the scalp might explain this discrepancy.

CMRO_2_ did increase during vasopressor-inotrope therapy, but the change was small and statistically insignificant. Adult animal models have shown diverging results, with unaffected CMRO_2_ in some studies [28] and increased CMRO_2_ in others [11]–[13], [29].

Heterogenic microvascular perfusion is associated with an increased median oxygen diffusion distance [13]. Consequently, an increase in the heterogeneity of cerebral perfusion – by vasopressor-inotrope therapy increasing flow in some microvascular territories but not in others – will leave the oxygenation in those vascular beds unchanged, and hence the average cerebral oxygenation at the regional level will be increased proportionally less. Overall, we demonstrated a statistical insignificant increase in anatomical heterogeneity and a statistical insignificant decrease in flow in capillaries compared to all vessels. We made use of a large number of tests to evaluate heterogeneity. This necessitates an overall view. Therefore, we do not ascribe significance to the fact that adrenaline apparently decreased anatomical heterogeneity for all vessels but not for capillaries. We are unaware of other studies investigating possible changes in cerebral microvascular heterogeneity during vasopressor infusion.

It is possible that the increases in cerebral metabolism and microvascular heterogeneity, although statistically insignificant, may explain the observed mismatch between cerebral perfusion and oxygenation. Alternatively, a change in the arterio-venous volume ratio as seen by NIRS [30], may explain it. To investigate this possibility, we used a manual iterative approach to estimate vasopressor-inotrope induced changes in the arterial-to-venous volume ratio that might explain the measured changes in cerebral oxygenation (OI) and cerebral blood volume (tHb). This approach demonstrated that the arterial-to-venous volume ratio should only change as little as 1% in order to be able to explain the observed mismatch (see Appendix S1).

Besides the already discussed methodological consideration regarding the NIRS technology, the main methodological considerations are as follows. Firstly, even though we studied a rather large and homogenous group of piglets that from a cerebral hemodynamic point of view stayed stable during the experiment, large inter-individual and intra-individual differences were observed. This, unfortunately, increases the likelihood of a type II error. Secondly, although newborn piglets have anatomical, physiological and developmental similarities with the human term infant [31], [32], it is difficult to extend results obtained in a piglet model to human infants. Thirdly, findings in normotensive animals might not correspond to those in animals with cardiovascular compromise [10]. Fourthly, we did not account for the fact, that the balloon-inflations induce a decrease in perfusion and oxygen delivery to the tissue distal to the balloon, which might trigger an autonomic feedback to the brain. However, as demonstrated in Fig. 2, the cerebral response to these stimulations did not change over a period of time. Furthermore, lactate did not accumulate in the piglets. So in other words, our model seems stable in spite of this limitation.

In conclusion, we demonstrated a moderate vasopressor-inotrope induced mismatch between cerebral perfusion and oxygenation. Removing the scalp did not matter, so vasopressor-inotropes appear to have direct cerebral actions. The statistically nonsignificant increases in cerebral metabolism and/or microvascular heterogeneity may explain the mismatch. Alternatively, it may simply reflect a vasopressor-inotrope-induced decrease in the arterial-to-venous volume ratio as detected by near-infrared spectroscopy. Given the large population of sick infants who are treated with vasopressor-inotropes, further studies to elucidate possible direct cerebral effects in the newborn brain are warranted.

## Supporting Information

Appendix S1 (DOCX)
