# Supplementary material for: Cerebral Effects of Commonly Used Vasopressor-Inotropes: A Study in Newborn Piglets
Source: PLoS One. 2013 May 20;8(5):e63069. doi: 10.1371/journal.pone.0063069 (PMC3659109; doi:10.1371/journal.pone.0063069)
Supplement: Appendix S1 — (DOCX) [file pone.0063069.s001.docx]

Appendix I: Quantitative interpretation of findings

We used a manual iterative approach to estimate concomitant changes in the arterial-to-venous volume ratio that might explain the measured changes in tHb ($tHb=O_{2}Hb+HHb$) and OI ($OI=\frac{O_{2}Hb-HHb}{2}$).

First, we used the measured blood hemoglobin (bHb) and an estimated cerebral blood volume (CBV) of 3 ml/100g, to estimate the cerebral hemoglobin concentration (cHb in µM monohaemoglobin) as:

$$cHb=CBV\times\frac{1.05}{100}\times bHb*\frac{1000}{4}\times CLVHR (I)$$

where factor 1.05/100 is used to convert CBV in ml/100 g to ml/ml, factor 1000/4 is used to convert mM monohaemoglobin to µM tetrahaemoglobin as seen by near-infrared spectroscopy and CLVHR is the cerebral to large vessels haematocrit ratio (equals 0.69) accounting for the use of large vessels bHb concentration.

Then, based on the measured arterial saturation (SaO_2_) and the venous saturation (SvO_2_) we estimated the relative changes in the arterial-to-venous volume ratio by means of a manual iterative process using equation (II) and (III):

$$O_{2}Hb=cHb\times\left( \left( SaO_{2}\times A_{frac} \right)+\left( SvO_{2}\times V_{frac} \right) \right) ( II)$$

$$HHb=cHb\times\left( \left( \left( 1-SaO_{2} \right)\times A_{frac} \right)+\left( \left( 1-SvO_{2} \right)\times V_{frac} \right) \right) (III)$$

where A_frac_ is the ´arterial volume´ divided by CBV and V_frac_ is the ´venous volume´ divided by CBV. The SvO_2_ was measured during pharmacologically induced changes in MAP and estimated during nonpharmacologically induced changes in MAP as:

$$SvO_{2}=SaO_{2}-\frac{1}{1+\Delta CBF} (IV)$$

where ΔCBF is change in cerebral blood flow pr change in MAP estimated by means of the measured percentage change in laser-Doppler flow. Equation (IV) assumes that cerebral metabolism is unchanged during nonpharmacologically induced changes in MAP.
